# Rural Adolescents: Parental Communication on Sexual and Reproductive Health Matters in Jimma Zone, Southwest Ethiopia

**DOI:** 10.1155/2022/8033853

**Published:** 2022-08-26

**Authors:** Gelila Abraham, Gebeyew Tsega Nebeb, Beshea Gelana Deressa, Berhane Megerssa, Negaligh Berihanu Bayou, Aderajew Nigusse Teklehaymanot, Kiddus Yitbarek Khali

**Affiliations:** ^1^Department of Health Management and Policy, Jimma University, Jimma, Ethiopia; ^2^Department of Population Health and Family Health, Jimma University, Jimma, Ethiopia

## Abstract

**Purpose:**

There are very limited evidences showing the status of adolescent-parent communication in rural areas of Ethiopia as most of the studies focus in urban areas and were school-based. Therefore, this study intends to determine the adolescent-parent communication on sexual and reproductive health matters and its determinants among rural adolescents in Jimma Zone, Southwest Ethiopia.

**Methods:**

Community-based cross-sectional study design was employed using the multistage sampling technique. Structured questionnaire was used to collect the data. The data was cleaned and entered into Epi data version 3.1 and exported to SPSS version 23 for descriptive and regression analysis.

**Results:**

From 833 adolescents participated in the study, only 364 (43.7%) of them had ever discussed sexual and reproductive health matters of which males represent 196 (53.8%) followed by females 168 (46.2%). Among these, only 35 (9.6%) had discussed with their mother, and 24 (6.6%) had discussed with their father. The proportion of adolescents who communicated with their parents on sexual and reproductive health issues was 364 (43.7%). Multivariable logistic regression analysis indicated that never getting information on SRH issues (AOR = 0.5, 95% CI, 0.4-0.8) and particularly on sexually transmitted infections (AOR = 0.5, 95% CI, 0.4-0.7) and unknowing the period in which there is a possibility to be pregnant for a girl (AOR = 0.2, 95% CI, 0.04-0.9) were found to be independent factors affecting adolescent-parental communication negatively.

**Conclusion:**

This study's result implies that the majority of the adolescents in the rural area were not communicating with their parents about sexual and reproductive health issues. However, most of them knew about different sexual and reproductive health services and where they could be found. Therefore, the provision of detailed information on the importance of communication with their parents on such a sensitive issue is suggested. Further research is needed to identify the barrier from the parents' side.

## 1. Introduction

The term “adolescent” is used to mean individuals between the age group 10 to 19 years. It is the second period of life in which an individual undertakes main physical and psychological changes [1]. Some 1.2 billion adolescent make up 18 percent of the world's population. In sub-Saharan Africa, adolescents aged 10-19 years make up the largest proportion (23%) of the region's population. Sub-Saharan Africa is the only region of the world in which the number of adolescents continues to grow significantly [2].

Although adolescents make up a large proportion of the population in the developing world, where most humanitarian disasters occur, their sexual and reproductive health (SRH) needs are largely unmet [3] . A sequence of multilayered barriers currently forbids good sexual and reproductive health for adolescents. Politically, low priority for adolescent and sexual reproductive health was observed, and even restrictive laws and policies are in place. Discussion on issues of ASRH is difficult due to different cultures, societies, and religion that are not enhancing the environment, and this is often demonstrated through the stigmatization of sexual health concerns, in particular STIs/HIV [4].

Young people are currently the group that were most severely impacted by HIV/AIDS. In 2009, young people aged between 15 and 24 years accounted for 41% of all new HIV infections among adults over the age of 15, and it is estimated that globally, more than five million young people aged 15-25 years were living with HIV. Most of these young people live in Sub-Saharan Africa, most are women, and most do not know their status [4] [5]. The global perspective showed that the huge regional variation indicates mainly social and economic determinants of sexual behavior, which calls for a comprehensive behavioral interventions if needed to have a respondent population to a given services [6].

Parents want their son and daughter to grow and develop in healthiness and able to contribute significantly to this world. To make this real and effective, the communication between parents and children is vital. However, many parents are unable to address the sensitive issues around youth-like, sexuality, and reproduction. The main reason may be their own knowledge, misconceptions, hopes, and fears [7]. The challenge is high in many sub-Saharan African countries as the social environment in several traditional communities still hinders such communication. Moreover, when adolescents feel unconnected to home, family, and school, they may become involved in activities that put their health at risk [8], but there are evidences showing that parent-adolescent communication on SRH matters was found to positively affect sexual behaviors amongst adolescents particularly in delaying sexual initiation, reduced sexual activity, and improved use of SRH services [9, 10].

Addressing parental adolescent communication about sexual and reproductive health issues through research will guide the development of appropriate strategy and intervention of recommendations to have healthy youth. Therefore, this study is aimed at assessing adolescent-parent communication on sexual and reproductive health matters among rural adolescents in Jimma Zone, Southwest Ethiopia.

## 2. Materials and Methods

### 2.1. Study Setting

The study was conducted in Jimma Zone, Southwest Ethiopia. Jimma Zone is one of the zones in Oromia regional state and is located at 335 km away from the capital of the country, Addis Ababa. It is divided into 20 districts and one town administration with a total of 548 kebeles (the smallest administrative unit) among which 515 (93.9%) are rural. Based on the 2007 Census, the projected total population of the zone was 3,209,127 in 2017 11. Though there was a remarkable effort made to increase accessibility of adolescent-friendly sexual and reproductive health services, more exertions are needed to touch the segment of the population particularly at rural areas. As a major strategy to expand primary health care coverage in the country, the Health Extension Program (HEP) was in place since 2003 and brought a significant change on the health of the population. Adolescent-friendly reproductive health service was among the services provided by the program under the subtheme family health, though the service lacks focus on adolescents because of different challenges like productivity of the health extension workers and other social determinants 12.

### 2.2. Study Design and Period

We used community-based cross-sectional study design from January 03 to 30, 2018.

### 2.3. Study Participants

The source population was all adolescents whose age ranges from 10 to 19 years living in the study area at the time of data collection. The study population included adolescents (10–19 years old) who had the chance of being randomly selected from the source population.

### 2.4. Sample Size Determination

Sample size was determined using the single population formula by considering assumptions of proportions of parent-adolescent communication on SRH matters to be 50% as there is no study conducted in rural setting at community level, desired precision of 5%, and 95% confidence level. We have considered a design effect of two and 10% nonresponse rate, and the final sample size was 845 adolescents in the age group 10-19 years.

### 2.5. Sampling Procedure

Series of sampling procedures were employed to select study subjects. First, multistage sampling procedure was employed to select representative sample of adolescents in the selected districts of the Zone. From a total of 20 districts, six were selected randomly and from the selected districts, six rural kebeles in each district were again selected randomly, making a total of 36 kebeles. Then, the calculated sample size of adolescents was allocated to the selected kebeles proportional to the total number of adolescents in the kebele. This information was sought from a family folder register located at the health posts in each kebele. After selecting households using systematic random sampling technique, one adolescent per the selected household was interviewed. In case of more than one adolescent found in a particular household, one of them was selected randomly for the interview. Adolescents who reside in the kebele for more than six months were included in the study.

### 2.6. Data Collection and Quality Procedures

Data were collected using a pretested structured questionnaire which was developed by reviewing related studies 13, 14. The questionnaire was first developed in English, translated into Afan Oromo and then retranslated back to English to check its consistency. We conducted a pretest on 5% of our sample size among adolescents residing other than the study setting. We revised the questionnaire based on the gaps identified during the pretest. We used an interviewer based data collection technique to gather the data. We recruited six data collectors and three supervisors and oriented for two days on how to run the data collection procedure. Informed verbal consent was obtained from adolescents aged 18-19 and for whose age was less than 18, and we sought assent and informed parental consent before the interview begin.

### 2.7. Study Variables

Data were collected on independent variables like sociodemographic, economic, sociocultural, participants' knowledge on SRH issues, and individual/personal factors related to reproductive health service including sex history. The dependent variable was adolescent-parent communication about sexual and reproductive health issues.

### 2.8. Operational Definitions

#### 2.8.1. Parents

They refer to a father or mother: one who begets or one who gives birth to or nurtures and raises a child or relative who plays a role of the guardian role.

#### 2.8.2. Adolescents

They refer to study participants in the age range of 10-19 years.

#### 2.8.3. Parental Communication

It refers to a discussion between adolescents and their parents on at least one SRH matter (e.g., contraception, STI, HIV/AIDS, sexual intercourse, unintended pregnancy, avoiding premarital sex, condom, changes during puberty, menstrual cycle, and which RH services are available) which were considered to have communicated on SRH matters.

### 2.9. Data Processing and Statistical Analysis

Data were edited, coded, and entered using EPI Info version 3.5.1 15 and then transported to SPSS window version 24.0 for statistical analysis 16. Descriptive data analysis was used to describe the distribution of factors for adolescent-parent communication about sexual and reproductive health issues. To ascertain the association between the dependent and independent variables, binary logistic regression analysis was used. We computed odds ratio with 95% CI to show the strength of the association between the outcome and independent variables. Variables with significant association (*P* value <0.05) in the bivariate analysis were entered into multivariate analysis to determine independent associated factors of adolescent-parent communication on SRH matters. Therefore, the independent effect of each explanatory variable on an outcome variable was determined, whereas controlled for others.

## 3. Results

### 3.1. Sociodemographic Characteristics of Respondents

Eight hundred thirty-three adolescents participated in the study making a response rate of 98.6%. Nearly all participants were in the age group of 15-19 (97%) and single (97.8%). Male and female were almost at equal proportion. Slightly more than three-fourth were in secondary school (77.31%) and Oromo in ethnicity (76.23%). Majority were Muslims (70.9%) and living with their parents (81.3%). Large segment of the adolescents reported that their parents (father and mother) have no formal education and are farmers (see Table 1).

### 3.2. Individual Factors on Sexual and Reproductive Health Services

#### 3.2.1. Adolescents' Knowledge on SRH Matters

Adolescents were asked which types of RH services they know, and the most known service was voluntary counseling and testing (VCT), 497 (83.5%) followed by condom, 404 (68.7%; other family planning services, 316 (53.1%); treatment of sexually transmitted illnesses 255 (42.9%); pregnancy test, 203 (34.1%); information and counseling, 167 (28.1%); abortion care service, 140 (23.5%); and lastly, antenatal care, 161 (27.1%).

Four hundred eighty-two (81%) adolescents know where these RH services are available, and the large majority reported government health facilities: health center (91.9%), hospital (63.9%), health post (58.1%), and 138 (28.6%) mentioned private health facilities. Six hundred and nine (73.1%) adolescent had heard symptoms of sexually transmitted illnesses, and the mostly heard were burning sensation during urination, 402 (66%); genital ulcers/sores, 341 (56%); and genital itching, 291 (47.8%), but only 92 (15.1%) had heard of lower abdominal pain.

More than three-fourth (83.6%) had reported that they know the period in which there is a possibility of becoming pregnant for a girl and while asked for their period: 307 (44%) replied just after menstruation, 280 (40.2%) replied during menstruation, 99 (14.2%) replied two weeks before menstruation, and the rest 13 (1.6%) replied always. Large majority of the adolescents (94.7%) believed that HIV/AIDS infection is the most important risk during penetrative sexual intercourse followed by other sexually transmitted illnesses (81.8%) and pregnancy (52.7%).

#### 3.2.2. Information Sources for SRH Matters

Five hundred ninety-five (71.4%) adolescent reported that they have seen/heard a message about SRH matters. Among mentioned sources of information, school and media (TV and radio) account for 44% and 23%, respectively. However, only 9% and less than 5% adolescents reported that friends and parent talk as source of SRH information.

#### 3.2.3. Adolescents' Sexual History

Ninety-seven (11.6%) adolescents had ever had sexual intercourse at least once in their life time. Among these, 76 (78.35%) were male, and the rest 21 (21.65%) were female adolescents. The mean age for sexual commencement was 16 years, and the SD was 1.5. Additionally, large majority of the adolescents reported that they had it with their boy/girlfriend for the first time.

Among those who had sexual intercourse, 56 (57.7%) of them had sexual intercourse in the past 12 months, those who had sex with one partner were 57 (58.76%), and with two and more partners were 40 (41.24%). Forty-one (73.2%) of those who had sexual intercourse in the last 12 months had made ever use of condom while 44 (45.36%) had used condom during their first time sex. Adolescents who did not used any condom ever reported the reason for not using a condom: partner trust (30.2%) and embarrassed to buy (11.6%).

### 3.3. Communication on SRH Matters

Three hundred sixty-four (43.7%) adolescent had ever discussed RH matters of which males represent 196 (53.8%) followed by females 168 (46.2%). Among these, only 35 (9.61%) had discussed with their mother, 24 (6.6%) had discussed with their father, 53 (14.5%) discussed with their brother/sister, 315 (86.5%) with their friends, and 27 (7.42%) with health care providers. While asked for the reasons for not discussing RH matters with their parents, adolescents replied as cultural and religious taboo, feeling ashamed and preferring friends for communications were among the major (see Table 2).

### 3.4. Factors Associated with Communication on SRH Matters

Bivariate analysis was conducted with sociodemographic variables, family factors, adolescent's knowledge on SRH matters, and sexual history of adolescents. *P* value <0.05 was considered significant for independent variables.

The result showed that none of the sociodemographic characteristics of adolescent was found to be associated with communication on SRH matters. The odds of discussing on SRH matters were 60% less in adolescent who did not ever get information on SRH matters than adolescents who ever got SRH information (COR = 0.4, 95% CI, 0.3-0.5). Adolescents who never read/heard of diseases that can be transmitted through sexual intercourse were 60% less likely to communicate on SRH matters than adolescent who ever read/heard of diseases that can be transmitted through sexual intercourse. The odds of discussing on SRH matters were 40% less in adolescent who never had sexual intercourse in their lifetime than adolescents who had ever sexual intercourse (COR = 0.6, 95% CI, 0.4-0.9). Adolescents who did not know the period in which there is a possibility of becoming pregnant for a girl were 80% less likely to communicate on SRH matters than adolescent who know the period in which there is a possibility of becoming pregnant for a girl is. All variables with *P* value ≤0.2 in the bivariate analysis were further analyzed by multivariate logistic regression mode (Table 3).

Results of the multiple logistic regression showed that ever getting SRH information, ever getting information on STI and knowing the period in which there is a possibility to be pregnant for a girl are factors significantly associated with adolescent-parent communication on SRH matters. Adolescents who never got information on SRH and specifically on STI were 50% less likely to discuss with parents than their counterparts (AOR = 0.5, 95% CI, 0.4-0.8 and AOR = 0.5, 95% CI, 0.4-0.7). Moreover, adolescents who did not know the period in which there is a possibility to be pregnant for a girl were 80% less likely to discuss on SRH matters with their parents (AOR = 0.2, 95% CI, 0.04-0.9) (see Table 3).

## 4. Discussion

This study determined the status of rural adolescent-parent communication on SRH matters. The findings from this study showed that nearly three fourth adolescents have information on any of SRH matters. Voluntary counseling and testing and contraceptives including condom were the most commonly known SRH services by adolescents. Moreover, large majority of the respondents mentioned governmental health facilities as a place to get SRH services. This could be due to the fact that governmental health facilities are most accessible to the large segment of the population, particularly for those residing in the rural area. Adolescents had first sexual intercourse at the mean age of 16 years old. Approximately three out of four adolescents had ever heard/read a message regarding SRH matters. Media (TV and radio) were the major sources of SRH information for rural adolescents. Respondents of the study revealed that cultural taboo and fear to talk with parents were the main reasons for not having discussion on SRH matters, which is in line with other studies conducted in other settings of the country 13, 17, 18.

The finding of this study showed that less than half of adolescents (43.7%) ever discussed on SRH matters with their parents which is in line with a study conducted in East Wollega Zone (42.5%)^17^ and Bale (47%)^19^ but higher than studies conducted in other settings 13, 20–23. This difference could be probably due to all studies that were institution-based, while ours were community-based which could represent all adolescents instead of their educational status. On the other hand, a study finding from Haiyk town, Northeast Ethiopia, semiurban town, was much higher than the finding of this study in which the parent-adolescent communication on SRH matters was 83%. This difference could be due to the nature of the study participants by which the former one focused on preparatory school students in semiurban setting whose knowledge and awareness could be better than adolescents in the rural community.

The results of multiple logistic regression models revealed that ever getting SRH information, ever getting information on STI, ever having sexual intercourse, and knowing the period in which there is a possibility to be pregnant for a girl showed significant positive association with communication on SRH matters.

Adolescent who never got information on SRH matters were 60% less likely to discuss on SRH than their counterparts and additionally, adolescents who never got information on sexually transmitted illnesses were 40% less likely to discuss on SRH matters which was supported by the findings of studies conducted in other parts of the country which showed that knowledgeable adolescents are more likely to discuss SRH matters with their parents 19, 21, 22. This could be due to the fact that adolescents who have an awareness and knowledge on the aspects of SRH will be eager to communicate on the issue and need to know more by discussing with their nearby people.

Adolescents who never had sexual intercourse were 40% less likely to communicate on SRH than adolescents who ever had sexual intercourse. This could imply that adolescents who practiced sexual intercourse may need to know more about their reproductive health either for the sake of using the available services or they have got a chance to talk on the issue with their sexual partner which will be easy for them to discuss on SRH matters, but adolescents who never had sexual intercourse in their lifetime will be afraid of talking on SRH matters due to different reasons including cultural taboo. There is a support finding from a study conducted in Woldia regarding this issue, which revealed that adolescents who ever had sexual intercourse were 1.7 times more likely to discuss SRH matters with their parents than adolescents who never had sexual intercourse 22. Adolescents who did not know the period in which there is a possibility to becoming pregnant for a girl were 80% less likely to communicate on SRH matters than their counterparts. This implies that low knowledge and awareness of adolescents have a direct effect on communication regarding SRH. Adolescent may fear to talk about the issue they did not know well and may prefer silence.

### 4.1. Strength and Limitation of the Study

The strength of this study is community-based to include all subgroups of adolescents, and the limitation is that it was based on interview which might be affected by social desirability bias as the interview contains sensitive issues barrier for open discussion.

## 5. Conclusion

The findings of this study revealed that adolescent-parent communication on SRH matters was found to be low. Adolescents prefer to discuss SRH issues with friends/peers rather than parents and even than brother/sister. Cultural taboo and fear were among the major reasons mentioned by adolescent not to have parental communication on SRH issues. Ever getting SRH information, ever getting information on STI, ever having sexual intercourse and knowing the period in which there is a possibility to becoming pregnant for a girl were the determinants of adolescent-parent communication on SRH matters. Promoting parent-adolescent communication on sexuality through tailored intervention needs to be applied to improve the commination. Moreover, it is recommended to have further studies on how adolescents and parents communicate on SRH issues as it may have a great impact about not having a good communication.

## Figures and Tables

**Table 1:** Sociodemographic characteristics of adolescents and their parents in Jimma Zone, Southwest Ethiopia, 2017 (*n* = 833).

Variables	Number (*n* = 833)	Percent
Age		
10-14	25	3
15-19	808	97
Sex		
Male	429	51.5
Female	404	48.5
Educational status		
No formal education	14	1.68
Primary (1–8)	126	15.2
Secondary (9, 10)	644	77.3
Preparatory (11, 12)	49	5.8
Ethnicity		
Oromo	635	76.3
Amhara	127	15.3
Guraghe	29	3.5
Others^a^	42	5.1
Marital status		
Single	815	97.8
Married	18	2.2
Religion		
Muslim	591	70.9
Orthodox	206	24.7
Protestant	34	4.1
Wakefata	2	0.3
Living arrangement		
With parents	677	81.3
With relatives	53	6.4
With friends in rented house	93	11.2
Alone	10	1.2
Educational status of father		
No formal education	392	47.1
Primary	358	42.9
Secondary	65	7.8
Higher education	18	2.2
Occupation of father		
Farmer	666	79.9
Small scale merchant	111	13.3
Government employee	29	3.5
Others^b^	27	3.3
Educational status of mother		
No formal education	490	58.8
Primary	304	36.5
Secondary	33	4.0
Higher education	6	0.7
Occupation of mother		
Farmer	642	77.1
Housewife	106	12.7
Merchant	47	5.6
Others^c^	38	4.6

^a^Others include Tigre and Kafa. ^b^Others include self-employee and daily laborer. ^c^Others include government employee, self-employee, and daily laborer.

**Table 2:** Reasons mentioned by adolescent for not discussing SRH matters with their parents, Jimma Zone, Southwest Ethiopia, 2017.

Reasons for not discussing (*n* = 519)	Frequency (percentage)
Cultural taboo	279 (53.7)
Preferring friends because of fear	167 (32.2)
Do not know	112 (21.6)
Feeling ashamed	89 (17.2)
Embarrassed	68 (13.1)

**Table 3:** Factors associated with adolescent-parent communication on SRH matters among rural adolescents in Jimma Zone, Southwest Ethiopia, 2017.

Variables	Had discussion		Crude OR	Adjusted OR
	Yes (%)	No (%)		
Had ever got SRH information				
Yes (595)	308 (51.7)	228 (38.3)	1.00	1.00
No (238)	56 (23.5)	113 (47.5)	0.4 (0.3, 0.5)^∗^	0.5 (0.4, 0.8)^∗∗^
Had ever got information on STI				
Yes (609)	305 (50.1)	228 (37.4)	0.4 (0.3, 0.5)^∗^	0.5 (0.4, 0.7)^∗∗^
No (224)	59 (26.3)	113 (50.4)		1
Ever had sexual intercourse				
Yes (97)	55 (56.7)	32 (32.9)	0.6 (0.4, 0.9)^∗^	0.7 (0.5, 1.2)
No (736)	309 (41.9)	309 (41.9)	1.00	1.00
Know the period in which there is possibility to be pregnant for a girl				
Yes (696)	362 (52.1)	329 (47.3)	1.00	1.00
No (137)	2 (1.5)	12 (8.7)	0.2 (0.04, 0.7)^∗^	0.2 (0.04, 0.9)^∗∗^

^∗^ *P* < 0.05 and ^∗∗^*P* < 0.01.

## Data Availability

All data related to the manuscript is available, and we can provide upon request.

## Abbreviations

AOR: Adjusted odds ratio
AIDS: Acquired immune deficiency syndrome
CI: Confidence interval
HIV: Human immune deficiency virus
SRH: Sexual and reproductive health
STI: Sexually transmitted infections
RH: Reproductive health.
